# Tissue factor is induced by interleukin-33 in human endothelial cells: a new link between coagulation and inflammation

**DOI:** 10.1038/srep25171

**Published:** 2016-05-04

**Authors:** Stefan Stojkovic, Christoph Kaun, Jose Basilio, Sabine Rauscher, Lena Hell, Konstantin A. Krychtiuk, Cornelia Bonstingl, Rainer de Martin, Marion Gröger, Cihan Ay, Wolfgang Holnthoner, Wolfgang Eppel, Christoph Neumayer, Ihor Huk, Kurt Huber, Svitlana Demyanets, Johann Wojta

**Affiliations:** 1 Department of Internal Medicine II, Medical University of Vienna, Austria; 2 Ludwig Boltzmann Cluster for Cardiovascular Research, Vienna, Austria; 3 Department of Vascular Biology and Thrombosis Research, Medical University of Vienna, Austria; 4 Core Facilities, Medical University of Vienna, Austria; 5 Department of Internal Medicine I, Clinical Division of Hematology and Hemostaseology, Medical University of Vienna, Austria; 6 Ludwig Boltzmann Institute of Experimental and Clinical Traumatology, AUVA Research Centre, Vienna, Austria; 7 Austrian Cluster for Tissue Regeneration, Vienna, Austria; 8 Department of Obstetrics, Medical University of Vienna, Austria; 9 Department of Surgery, Division of Vascular Surgery, Medical University of Vienna, Austria; 10 3rd Medical Department for Cardiology and Emergency Medicine, Wilhelminen Hospital, Vienna, Austria; 11 Department of Laboratory Medicine, Medical University of Vienna, Austria

## Abstract

Tissue factor (TF) is the primary trigger of coagulation. Elevated levels of TF are found in atherosclerotic plaques, and TF leads to thrombus formation when released upon plaque rupture. Interleukin (IL)-33 was previously shown to induce angiogenesis and inflammatory activation of endothelial cells (ECs). Here, we investigated the impact of IL-33 on TF in human ECs, as a possible new link between inflammation and coagulation. IL-33 induced TF mRNA and protein in human umbilical vein ECs and coronary artery ECs. IL-33-induced TF expression was ST2- and NF-κB-dependent, but IL-1-independent. IL-33 also increased cell surface TF activity in ECs and TF activity in ECs-derived microparticles. IL-33-treated ECs reduced coagulation time of whole blood and plasma but not of factor VII-deficient plasma. In human carotid atherosclerotic plaques (n = 57), TF mRNA positively correlated with IL-33 mRNA expression (r = 0.691, p < 0.001). In this tissue, IL-33 and TF protein was detected in ECs and smooth muscle cells by immunofluorescence. Furthermore, IL-33 and TF protein co-localized at the site of clot formation within microvessels in plaques of patients with symptomatic carotid stenosis. Through induction of TF in ECs, IL-33 could enhance their thrombotic capacity and thereby might impact on thrombus formation in the setting of atherosclerosis.

Tissue factor (TF) is the primary trigger of the blood coagulation and as such is believed to play a decisive role in the development of thrombotic complications of atherosclerosis such as acute myocardial infarction, unstable angina, and stroke^1^. TF is expressed in atherosclerotic plaques by vascular smooth muscle cells (SMCs), monocytes, macrophages and endothelial cells (ECs)^2^. In addition, coronary atherectomy specimens of patients with acute coronary syndromes demonstrated higher TF levels than those from patients with stable angina pectoris^3^. Moreover, extracellular TF is present on circulating microparticles (MPs), and levels of TF-bearing MPs are elevated in patients with cardiovascular risk factors such as diabetes, hypertension, obesity, and dyslipidaemia, as well as in patients with acute coronary syndrome^4^,^5^.

In addition to TF-dependent thrombin generation and fibrin formation, TF may play a role in the destabilization of atherosclerotic lesions due to coagulation-independent mechanisms, as TF induces migration of vascular SMCs, angiogenesis, activation of protease-activated receptors and an inflammatory response^5^,^6^,^7^. Upon activation, ECs express TF on their surface. Tumor necrosis factor (TNF)-α, interleukin (IL)-1β, lipopolysaccharide (LPS), thrombin, histamine, oxidized low-density lipoprotein, or vascular endothelial growth factor have been shown to increase TF expression in ECs^5^,^8^.

Tissue factor pathway inhibitor (TFPI) is the primary inhibitor of TF-initiated coagulation. ECs represent the main source of TFPI, and heparin and thrombin induce the release of EC-bound TFPI^9^,^10^. In atherosclerotic plaques TFPI co-localizes with TF^11^. Blockade of TF by local administration of TFPI was shown to decrease arterial thrombosis in atherosclerotic lesions^12^, and TFPI has inhibitory effects on both SMCs migration and proliferation^11^.

IL-33, a novel member of the IL-1-family of cytokines, is a ligand of the ST2 receptor^13^. Full-length, biologically active IL-33 is released either from necrotic or living cells during tissue damage through infection or trauma, or during mechanical strain, respectively^14^,^15^. Most studies have investigated immunomodulatory properties of IL-33 and found that IL-33 promotes both Th1 and Th2 immunity^13^,^16^,^17^. Accumulating evidence suggests a role for IL-33 in the modulation of inflammatory pathologies of the respiratory system, the gastrointestinal tract, and other inflammatory diseases such as arthritis and atherosclerosis^16^. IL-33 and ST2 are both expressed in human atherosclerotic plaques^18^. Moreover, IL-33 induces activation of ECs towards an inflammatory phenotype through the upregulation of IL-6, IL-8, monocyte chemoattractant protein (MCP)-1, vascular cell adhesion molecule (VCAM)-1, intercellular adhesion molecule (ICAM)-1 and endothelial (E)-selectin^18^,^19^. IL-33 also promotes angiogenesis and modulates the proteolytic capacity of ECs by inducing urokinase-type plasminogen activator (u-PA) and plasminogen activator inhibitor type-1 (PAI-1)^20^,^21^. Clinical data revealed that IL-33 serum levels were associated with mortality in patients with ST-elevation myocardial infarction (STEMI)^22^,^23^. In addition, our group previously showed that increased levels of circulating IL-33 after coronary stent implantation are associated with coronary in-stent restenosis^24^.

Despite experimental studies highlighting IL-33 as an endothelial cell activator,^18^,^19^,^20^,^21^ and clinical studies showing an association of IL-33 levels with thrombotic complications after plaque rupture,^22^,^23^ little is known about the possible effects of IL-33 on the thrombotic capacity of ECs. The aim of this study was to investigate the influence of the inflammatory cytokine IL-33 on TF expression and activity in human ECs. In addition, we studied whether an IL-33-induced procoagulant state of ECs could be translated into increased coagulation capacity of human blood. Finally, we investigated a possible association between IL-33 and TF expression in human atherosclerotic plaques.

## Results

### IL-33 upregulates TF and downregulates TFPI in human ECs

**Figure 1:**
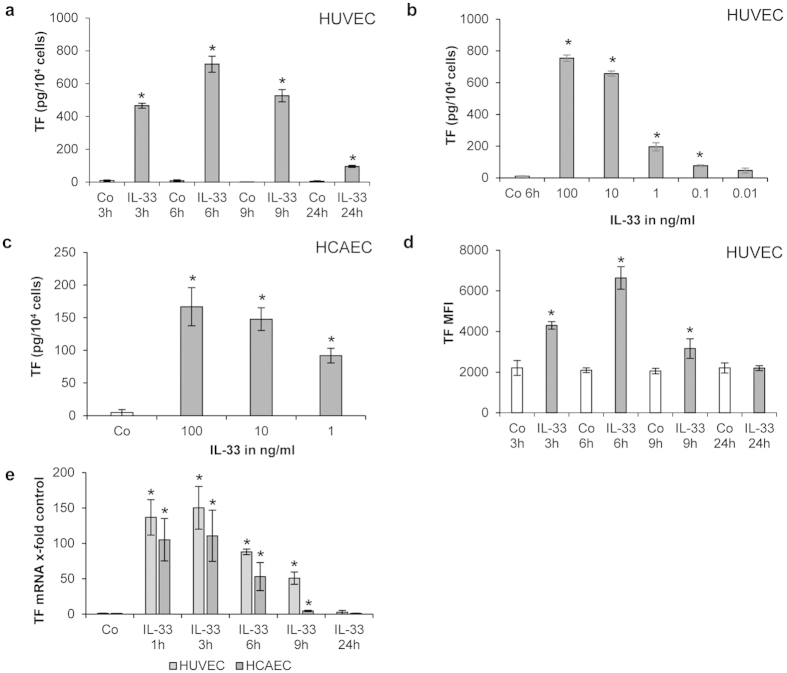
IL-33 increases TF protein and mRNA in human ECs in a time– and concentration-dependent manner. (**a,b**) HUVECs were incubated for 3, 6, 9 and 24 hours (h) in the absence (control, Co) or presence of rh IL-33 (100 ng/mL) (**a**) or HUVECs were incubated for 6 h without (Co) or with rh IL-33 at 100, 10, 1, 0.1 and 0.01 ng/mL (**b**). Cells were permeabilized with PBS containing 0.1% Triton X-100 and TF protein was determined; (**c**) HCAECs were incubated for 3 h without (Co) or with rh IL-33 at 100, 10, and 1 ng/mL. Cells were permeabilized with PBS containing 0.1% Triton X-100 and TF protein was determined. (**d**) HUVECs were incubated for 3, 6, 9 and 24 h without (Co) or with 100 ng/mL IL-33. TF expression at the cell surface was measured by means of flow cytometry; (**e**) HUVECs and HCAECs were incubated for 1, 3, 6, 9 or 24 h in the absence (Co) or presence of IL-33 (100 ng/mL). mRNA was prepared and Real Time PCR was performed. Values are given in pg/10^4^ cells (**a,b,c**), in mean fluorescence intensity (MFI) (**d**) or as TF/GAPDH mRNA x-fold change from respective control, which was set as 1 (**e**) and represent mean values ± standard deviation (SD) of three independent determinations. *p ≤ 0.05 compared to respective control.

We found that IL-33 significantly (p < 0.001) induced TF in both human umbilical vein ECs (HUVECs) and human coronary artery ECs, HCAECs (Fig. 1). Total cellular TF protein was increased in HUVECs lysates after 3, 6, 9 and 24 hours (h) of treatment with 100 ng/mL recombinant human (rh) IL-33 as compared to the respective control (Fig. 1a). A maximum 70-fold increase of TF protein production was seen after 6 h of incubation with 100 ng/mL rh IL-33 (Fig. 1a). This effect was also concentration-dependent as IL-33 at concentrations between 0.1–100 ng/mL significantly (p < 0.05) increased TF protein after 6 h in HUVECs (Fig. 1b). A significant (p < 0.05) concentration-dependent increase of TF protein expression was also detected in lysates of HCAECs after treatment with 1, 10, and 100 ng/mL rh IL-33 (Fig. 1c). Flow cytometry analysis showed significantly higher TF cell surface levels (p < 0.05) in HUVECs after 3, 6 and 9 h of treatment with 100 ng/mL rh IL-33 as compared to the respective control, with a maximum TF cell surface level reached again after 6 h (Fig. 1d).

As determined by Real Time-PCR, TF mRNA expression was strongly induced by 100 ng/mL rh IL-33 after 1, 3, 6 and 9 h in HUVECs and HCAECs (Fig. 1e). A maximum increase of TF mRNA expression of up to 150-fold or up to 110-fold was seen in HUVECs and HCAECs, respectively, after 3 h of treatment with rh IL-33 (Fig. 1e).

TFPI protein levels slightly but significantly (p < 0.05) declined in supernatants of HUVECs after 24 h and 36 h of treatment with 100 ng/mL rh IL-33 (Supplementary Fig. S1a). This effect of IL-33 was confirmed at the mRNA level as IL-33 at 100 ng/mL significantly (p < 0.05) reduced TFPI mRNA expression at 24 h and 36 h of treatment (Supplementary Fig. S1b).

### IL-33-induced TF expression is ST2- and NF-κB-dependent, but IL-1-independent

**Figure 2:**
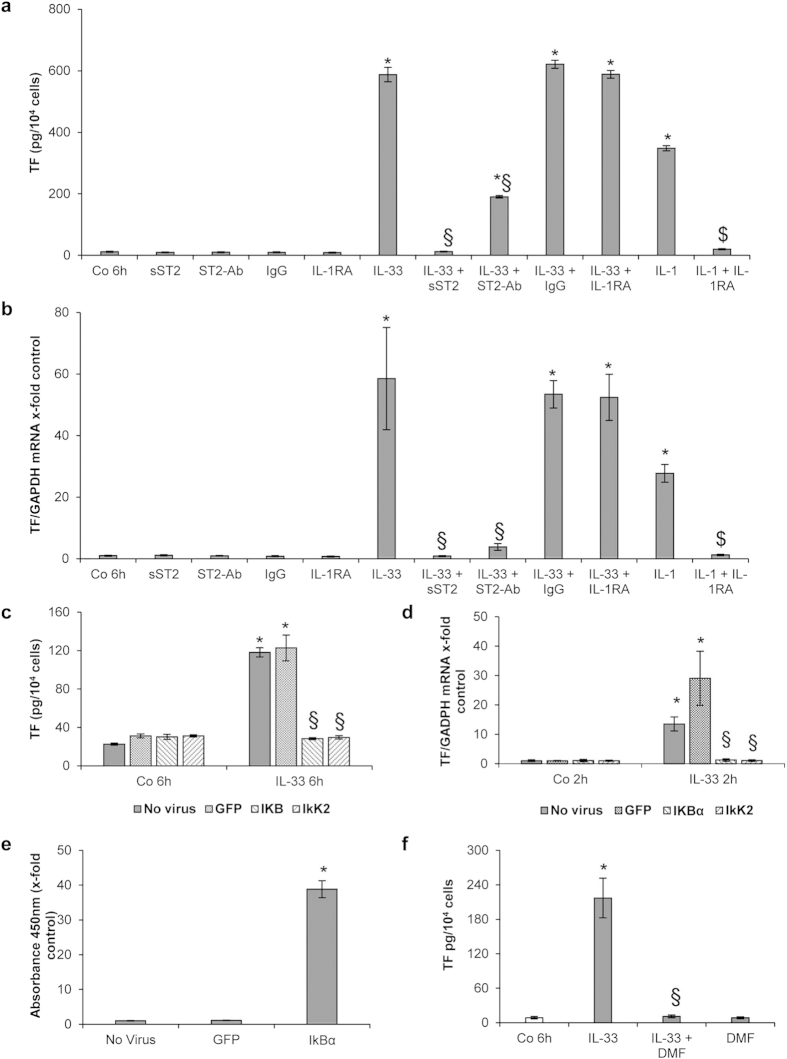
IL-33-induced TF expression is ST2- and NF-κB-mediated in HUVECs. (**a**,**b**) HUVECs were left untreated (Co), or incubated for 6 h with rh IL-33 (10 ng/mL) in the presence or absence of rh ST2/IL-1 R4 Fc chimera (sST2; 5 μg/mL), rh immunoglobulin G_1_ Fc (IgG; 5 μg/mL) or IL-1 receptor antagonist (IL-1RA; 10 μg/mL). In addition, IL-1β (100 U/mL) was added to the cells for 6 h with or without 10 μg/mL IL-1RA. Additionally, cells were pre-incubated for 30 min with 1 μg/mL human ST2/IL-1R4 antibody (ST2-Ab), before addition of 10 ng/mL IL-33. Cells were permeabilized with PBS containing 0.1% Triton X-100 and TF protein was determined (**a**), or mRNA was prepared and Real-Time PCR was performed (**b**). (**c–e**) HUVECs were left uninfected or were infected with adenoviral vectors for overexpression of IκBα (AdV-IκBα) or a dominant negative form of IκB kinase 2 (AdV-dnIkK2) or with a control adenovirus (AdV-green fluorescent protein (GFP)) for 4–6 h. 48 h post infection cells were treated with IL-33 (1 ng/mL) for 2 and 6 h whereas control cells were left untreated. Cells were permeabilized with PBS containing 0.1% Triton X-100 and TF protein (**c**) or IkBα protein (**e**) was determined, or Real-Time PCR for TF and GAPDH mRNA was performed (**d)**.(**f**) HUVECs were treated with medium alone (control, Co), IL-33 alone (10 ng/mL), dimethyl fumarate (DMF) at 100 μM alone or together with IL-33 for 6 h. Cells were permeabilized with PBS containing 0.1% Triton X-100, and TF protein was determined. Values are given in pg/10^4^ cells (**a**,**c**,**f**) as x-fold absorbance at 450 nm as compared to control (no virus; (**e**)) or as TF/GAPDH mRNA x-fold change from respective control, which was set at 1 (**b**,**d**) and represent mean values ± SD of three independent determinations. *p ≤ 0.05 compared to respective control; §p ≤ 0.05 compared to respective IL-33-treated cells; $p ≤ 0.05 compared to respective IL-1β-treated cells.

We and others showed previously that IL-33 exerts its effects via binding to its cell surface receptor ST2^13^,^18^,^21^. When HUVECs were incubated with both IL-33 at 10 ng/mL and the soluble extracellular domain of ST2 coupled to the Fc fragment of human IgG_1_ (sST2) for 6 h, the stimulatory effect of IL-33 on TF protein levels (Fig. 2a) and mRNA expression (Fig. 2b) was completely inhibited. This indicated that the increased TF production was a specific effect of IL-33 on HUVECs, which can be blocked by sST2. In order to investigate if TF induction by IL-33 was also ST2-mediated, we incubated ECs with a specific anti-ST2 antibody in the presence or absence of rh IL-33. The antibody against ST2 inhibited IL-33-induced TF protein levels (Fig. 2a) and mRNA expression (Fig. 2b) in HUVECs.

TF production may also depend on the autocrine action of IL-1, which has been shown to be upregulated in ECs by IL-33^18^. We showed here that IL-1 receptor antagonist, IL-1RA (10 μg/mL) had no effect on IL-33-induced TF protein production (Fig. 2a) and mRNA expression (Fig. 2b) in HUVECs. Conversely, the same concentration of IL-1RA completely inhibited IL-1-induced TF protein production and mRNA expression.

Upon binding to cell surface ST2, IL-33 activates the NF-κB pathway^13^,^18^,^21^. Using adenoviral constructs we could show here that overexpression of IκB α (AdV-IκBα) or overexpression of mutant dominant negative IκB kinase 2 (AdV-dnIKK2) abolished the IL-33-induced TF protein production (Fig. 2c) and mRNA expression (Fig. 2d) in HUVECs as compared to AdV-green fluorescent protein (GFP), used as a control adenovirus. As can be seen in Fig. 2e, HUVECs infected with AdV-IκBα had a 38-fold increase (p < 0.05) of IκBα protein levels as compared to cells infected with GFP or control cells demonstrating robust and efficient overexpression of IκBα in these cells. In addition, the NF-κB inhibitor dimethyl fumarate (DMF) at 100 μM abrogated IL-33-induced TF protein production in HUVECs lysates (Fig. 2f).

### IL-33 increases TF activity of ECs and EC-derived MPs

**Figure 3:**
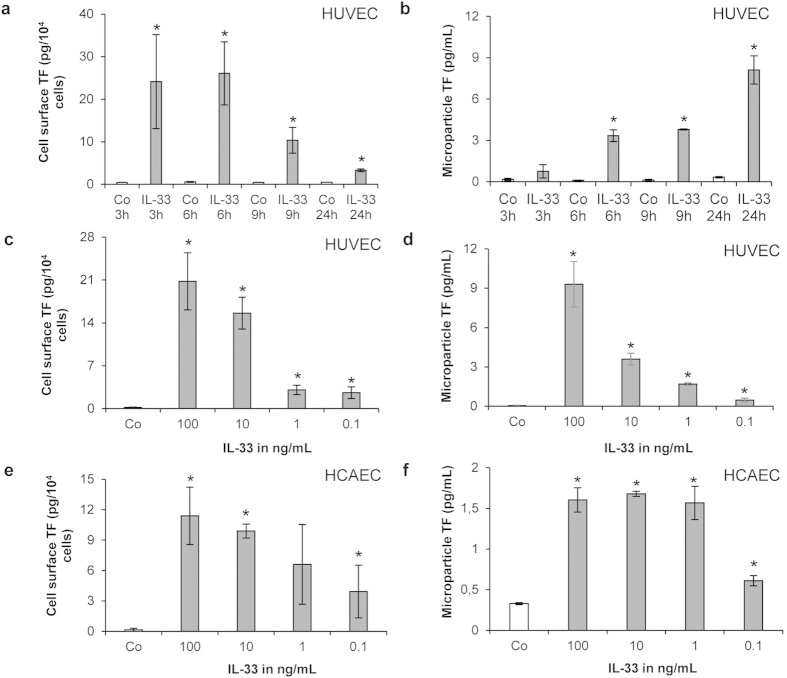
IL-33 increases TF activity of ECs and EC-derived MPs. (**a**,**b**) HUVECs were incubated for 3, 6, 9 and 24 h in the absence (Co) or presence of rh IL-33 (100 ng/mL), and TF activity was measured at the surface of HUVECs (**a**) or in MPs isolated from cell culture supernatants (**b**). (**c**,**d**) HUVECs and (**e**,**f**) HCAECs were incubated for 6 h without (Co) or with rh IL-33 at 100, 10, 1, and 0.1 ng/mL and TF activity at endothelial cell surface (**c**,**e**) or in MPs isolated from the respective cell culture supernatants (**d**,**f**) was measured. Values are given in pg/10^4^ cells (**a**,**c**,**e**) or in pg/mL (**b**,**d**,**f**) and represent mean values ± SD of three independent determinations. *p ≤ 0.05 compared to respective control.

At a concentration of 100 ng/mL IL-33 significantly (p < 0.01) increased TF activity on HUVECs surface between 3 and 24 h of treatment with a maximum effect reached after 6 h (Fig. 3a). When HUVECs were incubated with 100 ng/mL rh IL-33 for 3, 6, 9 and 24 h, we observed a time-dependent increase in TF activity on MPs derived from the supernatant of such treated cells, as compared to TF activity on MPs derived from the supernatant of untreated cells (Fig. 3b). Furthermore, IL-33 at concentrations ranging from 0.1–100 ng/mL also induced a significant concentration-dependent increase of TF activity at the surface of both HUVECs (Fig. 3c) and HCAECs (Fig. 3e). In parallel, MPs isolated from supernatants of such treated HUVECs (Fig. 3d) and HCAECs (Fig. 3f) also demonstrated a significant (p < 0.01) concentration-dependent increase in TF-activity with rh IL-33 at 0.1–100 ng/mL.

### IL-33-treated ECs reduce TF-dependent coagulation time of human whole blood and plasma

**Figure 4:**
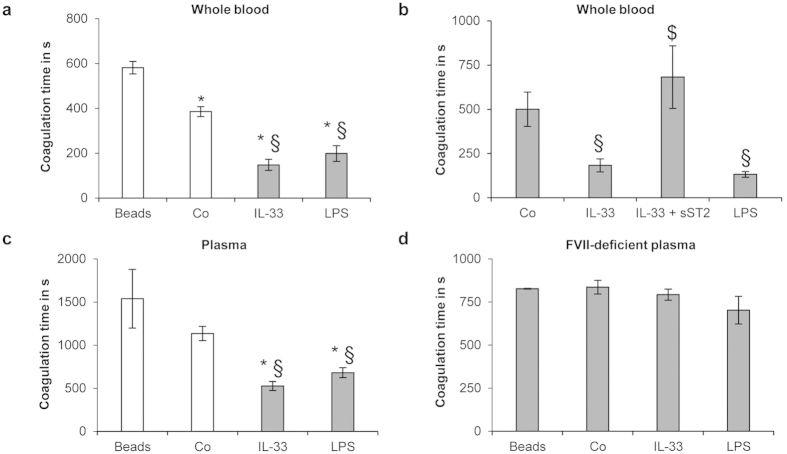
IL-33-treated ECs reduce coagulation time of human whole blood and plasma. HUVECs-coated micro-beads were incubated for 4 h in the absence (Co) or presence of rh IL-33 (100 ng/mL) or LPS (1 μg/mL). Uncoated micro-beads (beads) were used as negative control. In addition, HUVECs-coated micro-beads were incubated for 4 h with rh IL-33 (100 ng/mL) alone or together with sST2 (5 μg/mL). Such HUVECs-coated or uncoated micro-beads were added to citrated human whole blood (**a**,**b**), plasma from healthy volunteers (**c**) or factor VII (FVII)-deficient plasma (**d**) and coagulation time was measured. Values are given in seconds (s) and represent mean values ± SD of three independent determinations. *p ≤ 0.001 compared to uncoated micro-beads; §p ≤ 0.001 compared to respective control; $p ≤ 0.05 compared to IL-33-treated HUVECs-coated micro-beads.

In order to investigate whether the IL-33-induced increase of TF activity in ECs could affect the coagulation capacity of human blood, we performed thromboelastometry analysis, as described previously^25^. When HUVECs grown on micro-beads were treated with IL-33 and added to whole blood of healthy volunteers the coagulation time (CT) was significantly (p < 0.05) shorter as compared to untreated HUVECs on micro-beads or micro-beads without cells being added to whole blood (Fig. 4a). The effect on CT seen when HUVECs grown on micro-beads were treated with 100 ng/mL rh IL-33 was similar to the reduction of CT seen with HUVECs-coated micro-beads treated with 1 μg/mL LPS used as a positive control (Fig. 4a). In contrast, the extracellular domain of ST2 coupled to the Fc fragment of human IgG_1_ (sST2) reversed this IL-33-induced CT reduction (Fig. 4b). Furthermore, IL-33-treated HUVECs-coated micro-beads reduced the CT of human plasma from healthy volunteers as compared to untreated HUVECs-coated micro-beads or uncoated micro-beads (Fig. 4c). Conversely, IL-33-treated HUVECs-coated micro-beads did not reduce the CT of Factor (F) VII-deficient human plasma, suggesting that IL-33-induced CT-reduction is TF/FVII/activated FVII (FVIIa)-complex mediated (Fig. 4d).

### IL-33 and TF expression correlate in human atherosclerotic plaques

**Figure 5:**
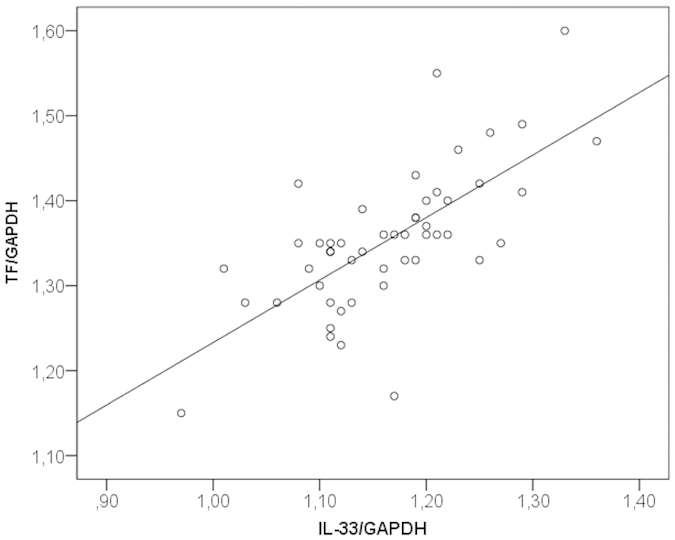
IL-33 and TF mRNA levels correlate in human carotid atherosclerotic plaques. RNA was isolated from human carotid atherosclerotic plaques (n = 57) and IL-33, TF and GAPDH mRNA were determined by RealTime-PCR. mRNA levels of TF were correlated with mRNA levels of IL-33 after adjustment for GAPDH.

**Figure 6:**
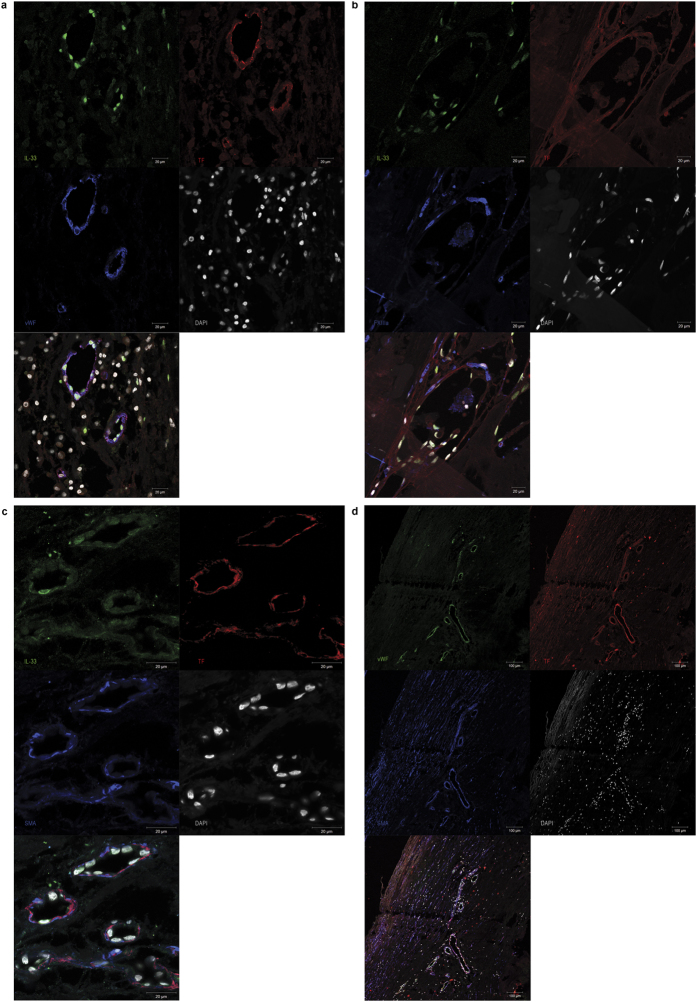
IL-33 and TF protein are co-localized in microvessels of human carotid atherosclerotic plaques. **(a**–**d**) Staining for IL-33 (green), TF (red), vWF (blue in **a**, green in **d**), FXIIIa (blue in **b**) and α-SMA (blue in **c**,**d**). Original magnification ×63 (**a**–**c**) or ×10 (**d**). Staining was performed with atherosclerotic samples from 4 different donors. Representative pictures are shown.

Human carotid atherosclerotic plaques were collected from patients with carotid stenosis undergoing carotid endarterectomy. Out of 57 plaque samples used for mRNA isolation, IL-33 mRNA and TF mRNA were simultaneously detectable in 50 samples. In those human carotid atherosclerotic plaques, IL-33 mRNA and TF mRNA expression levels showed significant positive correlation (r = 0.691, p < 0.001) (Fig. 5). Additionally, using immunofluorescence, IL-33 and TF protein was detected in human carotid atherosclerotic plaques (n = 4) where they co-localized with von Willebrand factor (vWF), demonstrating expression of both IL-33 and TF by ECs (Fig. 6a). Furthermore, IL-33 and TF protein were co-localized with FXIIIa at the site of clot formation within microvessels in atherosclerotic plaques from patients with symptomatic carotid artery stenosis (Fig. 6b). TF was also co-localized with α-smooth muscle actin (SMA) in the vessel wall (Fig. 6c,d), demonstrating strong expression of TF by SMCs. The specificity of the staining was confirmed by the use of respective isotype controls (Supplementary Fig. S2).

## Discussion

Inflammation exists in a mutually dependent association with coagulation, and both processes contribute decisively to the formation of atherosclerotic lesions and the development of atherothrombotic complications seen clinically as acute myocardial infarction, unstable angina, or stroke^26^,^27^. Unperturbed ECs interfere with inflammation and haemostasis by preventing cell adhesion to the vascular wall and by expressing antithrombotic and fibrinolytic molecules. Activated ECs on the other hand are known to support inflammation and to facilitate blood coagulation by expressing adhesion molecules, by down regulating antithrombotic proteins, by expressing procoagulants and by releasing procoagulant membrane vesicles^28^,^29^. ECs do not express TF under physiological conditions but several inflammatory stimuli, such as LPS or the “classical” cytokines IL-1β and TNF-α increase the procoagulant activity of ECs through induction of TF expression in these cells^30^,^31^,^32^.

Here, we show for the first time that IL-33, a member of the IL-1 cytokine family^13^, significantly increased TF mRNA expression, total cellular production of TF protein, as well as TF protein and activity on the surface of HUVECs and HCAECs *in vitro*. The time course of the IL-33-dependent endothelial TF production in our study was comparable to the effects of IL-1β and TNF-α on TF described previously^31^,^32^. The effect of IL-33 on TF protein production and TF activity was also concentration-dependent with significant effects seen at concentrations of >0.1 ng/mL in both HUVECs and HCAECs. This increase of TF activity correlated with the time course of TF cell surface levels observed by flow cytometry. Recently we have measured IL-33 serum concentrations of 0.1–0.7 ng/mL in patients with non-STEMI and STEMI^22^. One could speculate that local tissue concentrations of IL-33 could even be higher and thus could modulate TF expression and activity in ECs. Thus, we postulate here that IL-33 is another inflammatory cytokine, which can affect the thrombotic capacity of endothelial cells through stimulation of TF production.

Additionally we could show that IL-33 also increased TF activity on MPs isolated from ECs supernatants in a time- and concentration-dependent manner. TF-bearing MPs can be released from ECs, monocytes/macrophages, and SMCs^33^, and were detected in patients with atherosclerosis^4^. Such TF-bearing MPs were also shown to induce ECs proliferation^34^.

IL-33 signals through a heterodimeric receptor complex comprising the transmembrane receptor ST2 and the IL-1 receptor accessory protein^13^. ECs from both human umbilical vein and human coronary artery express ST2^35^,^36^. In this study, we showed that an antibody against ST2 inhibited IL-33-induced TF mRNA and protein expression. In addition, we show that sST2 abrogated the IL-33-induced production of TF in human ECs. These results suggest that the effects described here are specific for IL-33 and mediated through its interaction with ST2 in ECs. Upon binding to cell surface ST2, IL-33 activates the NF-κB-pathway^13^. We showed previously that IL-33 induced the upregulation of adhesion molecules, MCP-1, u-PA and PAI-1 in an NF-κB-dependent manner in ECs^18^,^21^. In the present study, the effect of IL-33 on TF was abolished by adenovirus-mediated overexpression of IkBα and a dominant negative mutant of IKK2 as well as by the NF-κB inhibitor DMF^35^,^37^. Thus, IL-33 induces TF expression in ECs via the activation of the NF-κB pathway.

As described above, IL-1β has also been shown to upregulate TF expression in ECs^38^,^39^. Since IL-33 can also induce IL-1β in these cells^18^, we have investigated if the induction of TF is indeed caused directly by IL-33. The effect of IL-33 on TF was independent of possible autocrine or juxtacrine effects of IL-1, as IL-1RA, which inhibits IL-1 binding to specific receptors, did not affect the IL-33-induced expression of TF mRNA or protein in ECs but inhibited the upregulation of TF by IL-1 in these cells.

Employing a thromboelastometry assay, we have investigated whether the IL-33-induced TF activity of ECs has an effect on coagulation of human whole blood and plasma. Our data show that IL-33-treated HUVECs-coated micro-beads significantly reduced the CT of human whole blood and plasma samples. This reduction in CT by IL-33 was comparable to the effect of LPS, which was used as a positive control. sST2 completely reversed IL-33-induced CT reduction in human whole blood, suggesting that the effect of IL-33 on CT was due to its specific interaction with ST2. In FVII-deficient plasma, IL-33-treated HUVECs-coated micro-beads had no effect on CT, suggesting that the observed reduction in CT is due to an increase of TF activity on the surface of ECs. Thus, the IL-33-induced increase of TF activity in ECs is able to affect the coagulation capacity of human whole blood and plasma.

In addition to induction of TF expression and activity, we could show here that TFPI mRNA and protein are down regulated by IL-33 in human ECs. Low TFPI levels are risk factor for both arterial and venous thrombosis^11^,^40^. Thus, IL-33 affects the procoagulant capacity of ECs not only by upregulating TF, but also by downregulating its inhibitor TFPI, thus shifting the balance towards a prothrombotic state.

A recent study has demonstrated an association of circulating IL-33 levels with the progression of carotid atherosclerotic plaques in rheumatoid arthritis patients^41^. Here we detected co-localization of IL-33 and TF protein in ECs of microvessels in human carotid atherosclerotic tissue. Moreover, IL-33 and TF protein co-localized with FXIIIa at the site of clot formation seen after intra-plaque haemorrhage in atherosclerotic plaques of patients with symptomatic carotid artery atherosclerosis. In addition, we found a positive correlation between IL-33 mRNA and TF mRNA levels in these lesions.

Previous studies have proposed an atheroprotective role of IL-33. Injection of high doses of murine IL-33 to apolipoprotein E knockout (ApoE^−/−^) mice fed a high-fat diet reduced atherosclerotic lesions in the thoracic aorta via increased IL-5 and oxidized low density lipoprotein auto-antibody production as well as by decreased macrophage foam cell formation^36^,^42^. IL-33 administration increased Th2 cytokines IL-4, IL-5, and IL-13, and reduced the Th1 cytokine interferon-γ by lymph node cells *in vitro* and in serum *ex vivo*^36^. In contrast, in a recent study, deficiency of IL-33 and ST2 did not affect development of atherosclerosis in ApoE-deficient mice^43^. We and others, however, showed that by inducing endothelial activation via increased expression of VCAM-1, ICAM-1, E-selectin, and augmented production of IL-6, IL-8, MCP-1, u-PA and PAI-1, IL-33 promotes the adhesion of human leukocytes to human ECs, modulates proteolysis of ECs and stimulates angiogenesis^18^,^19^,^21^,^44^. All these processes are detrimental in the development and progression of atherosclerotic plaques^6^. However, it should be emphasized that these effects are caused by the binding of extracellular IL-33 to the cell surface ST2 receptor. Possible effects of nuclear IL-33 remain to be further elucidated. Ali *et al.* proposed that nuclear IL-33 sequesters nuclear NF-κB and reduces NF-κB-triggered gene expression and thus dampens pro-inflammatory signalling^45^. Interestingly, a recent publication showed that knockdown of IL-33 by siRNA did not affect the sensitivity of endothelial cells towards IL-33^44^. In support of our findings, clinical studies demonstrated association of circulating IL-33 levels with both coronary and carotid atherosclerosis^22^,^23^,^24^,^41^. Given these results obtained in mice and humans, it should be emphasized that species-specific differences in the expression and regulation of IL-33 have been reported recently^46^,^47^. In particular it should be noted that while IL-33 was not detected in murine blood vessels, human vascular cells and in particular endothelial cells, strongly express IL-33^18^,^21^,^35^,^46^.

In summary, we showed here that the pro-inflammatory cytokine IL-33 augments the coagulatory capacity of human ECs through the increase of TF production and activity as well as the release of TF-bearing MPs by these cells. Thereby, IL-33 could contribute to the development of a prothrombotic microenvironment favouring thrombus formation at sites of vascular injury and inflammatory activation in blood vessels affected by atherosclerosis.

## Materials and Methods

### Cell culture

HCAECs and HUVECs were isolated, characterized and cultivated as described previously by us^18^,^48^. Cells used in this study were between passage 2 and 5 with a seeding density of 2 × 10^4^ cells/cm^2^ if not otherwise specified. The total cell number was counted with a haemocytometer after trypsinization. The Ethic Committee of the Medical University of Vienna, Austria, has approved the study and all study subjects have given informed consent. Experiments were carried out in accordance with the approved guidelines.

### Atherosclerotic tissue sampling

Atherosclerotic plaques were collected from 61 patients undergoing carotid endarterectomy (75.4% male, mean age 70.9 ± 8.1 years, 42.6% symptomatic, 70.2% with carotid artery stenosis of >90%). The carotid endarterectomy samples for IL-33 mRNA and TF mRNA analysis (n = 57) were snap-frozen in liquid nitrogen in the surgery room and stored at −80 °C until RNA extraction was performed. The carotid endarterectomy samples for immunofluorescence analysis (n = 4) were fixed in 4% formalin and embedded in paraffin. The Ethic Committee of the Medical University of Vienna, Austria, has approved the study and all study subjects have given informed consent.

### Treatment of cells

HCAECs and HUVECs were incubated in minimum essential medium 199 (M199, Sigma, St. Louis, MO, USA) containing 1.25% fetal calf serum (FCS, Cambrex, East Rutherford, NJ, USA), without or with rh IL-33 (R&D Systems, Minneapolis, MN, USA) at concentrations between 0.01 ng/mL and 100 ng/mL for time periods between 1 and 48 h. For blocking the transmembrane receptor ST2, HUVECs were pre-incubated for 30 min with 1 μg/mL monoclonal mouse anti-human ST2/IL-1R4 antibody and 10 ng/mL of rh IL-33 was added for 6 h. For soluble receptor inhibition experiments, HUVECs were incubated with IL-33 (10 ng/mL) alone or together with rh ST2/IL-1 R4 Fc chimera (sST2; 5 μg/mL, R&D Systems) for 6 h. rh immunoglobulin G_1_ Fc (IgG; 5 μg/mL, R&D Systems) was used as an isotype control. In order to determine whether the effect of IL-33 is dependent on IL-1β, HUVECs were cultured for 6 h in the absence or presence of IL-33 (10 ng/mL) or rh IL-1β (100 U/mL; R&D Systems) with or without IL-1RA (10 μg/mL; R&D Systems), respectively. In additional experiments, HUVECs were treated with the NF-κB inhibitor DMF (Sigma) at a concentration of 100 μM alone or together with IL-33 at 10 ng/mL for 6 h^35^,^37^. The culture supernatants were collected followed by removal of cell debris by centrifugation and stored at −80 °C until used. For analysis of total cellular TF protein levels, HUVECs were permeabilized with PBS containing 0.1% Triton X-100 (Sigma).

### Protein assays

TF protein levels in cell lysates were determined using a specific ELISA (Human coagulation Factor III/Tissue Factor Quantikine^®^ ELISA, R&D Systems). The TF ELISA measures recombinant and native human TF. The mean minimum detection limit was 0.69 pg/mL. TFPI protein levels were determined in cell culture supernatants with a specific ELISA (Human TFPI Quantikine^®^ ELISA, R&D Systems). The TFPI ELISA measures recombinant and native human TFPI. The minimum detection limit was 2.17 pg/mL.

### TF activity assay

The procoagulant activity of TF on the surface of ECs and TF activity of ECs-derived MPs upon stimulation with IL-33 was assessed by the ability of cells to convert FX to activated FX (FXa) in the presence of exogenously provided FVIIa, as described previously^49^. Briefly, HUVECs were seeded into 96-well plates at a seeding density of 1 × 10^4^ cells/well and incubated overnight. Cells were then treated with 100 ng/mL rh IL-33 for 3, 6, 9 and 24 h. After the incubation, supernatant was collected, cells were washed twice with HBSS containing BSA (HBSA) and 100 μl of HBSA was added into the wells. Samples were incubated with either mouse anti-human TF antibody (1 μg/mL; Becton Dickinson Biosciences Pharmingen, San Diego, CA, USA) or a control antibody (mouse IgG: 1 μg/mL; Sigma) for 15 min at room temperature. Next, 50 μL of HBSA containing 10 nM FVIIa, 300 nM FX and 10 mM CaCl_2_ was added to each well and the mixture was incubated for 2 h at 37 °C. FXa generation was stopped by the addition of 25 μL of 25 mM EDTA HBSA buffer. 25 μL of the chromogenic substrate S2765 (4 mM) was then added and incubated at 37 °C for 15 min. Finally, absorbance was measured at 405 nm. TF activity was calculated by reference to a standard curve generated using relipidated rh TF (0–55 pg/mL). The TF-dependent FXa generation was determined by subtracting the amount of FXa generated in the presence of TF antibody from the amount of FXa generated in the presence of the control antibody.

To investigate the TF activity of ECs-derived MPs, HUVECs were seeded into 96-well plates at a seeding density of 1 × 10^4^ cells/well and incubated overnight. Cells were then treated with 100 ng/mL rh IL-33 for 3, 6, 9 and 24 h. Cell culture supernatants were collected, and MPs were isolated by centrifugation at 20.000 g for 15 min at 4 °C, washed twice, and TF activity of ECs-derived MPs was assessed as described above^49^.

### Flow cytometry

HUVECs were incubated with rh IL-33 at 100 ng/mL as described above for 3, 6, 9 and 24 h. ECs were gently detached by detachment buffer (25 mM HEPES, 10 mM EDTA in Dulbecco’s PBS without calcium and magnesium, pH 7.4). Cells were then incubated with monoclonal antibodies directed against human TF (anti-human CD142 APC, clone HTF-1, eBioscience, San Diego, CA, USA) at a final concentration of 10 μg/mL. The cells were washed twice with PBS and resuspended in fixative solution (FACS flow solution, distilled water, BD CellFix^TM^). Bound fluorescence was analysed with a BD FACS Canto II using BD FACS Diva Software (BD Biosciences). Mean fluorescence intensities (MFI) for treated ECs were compared to the MFI of untreated cells.

### RNA purification and cDNA preparation

Total cellular RNA was isolated using RNeasy^®^ Mini Kit (Quiagen, Valencia, CA, USA). For tissue RNA isolation, the representative sample was collected from each carotid plaque. Frozen tissue was homogenized using a ball mill (Retsch, Haan, Germany), and mRNA was isolated using RNeasy^®^ Mini Kit (Quiagen). The total RNA amount was measured using a NanoDrop photometer (Thermo Scientific, Barrington, IL, USA). Reverse transcription was performed using GoScript™ reverse transcription system (Promega, Madison, WI, USA), according to the manufacturer’s instructions.

### Real-Time polymerase chain reaction

Real-Time-PCR was performed using LightCycler^®^ TaqMan^®^ Master (Roche, Basel, Switzerland). Primers were designed using the Roche Universal ProbeLibrary Assay Design Centre (http://www.universalprobelibrary.com/) (Supplemental Table I). Data were analysed using LightCycler Software Version 3.5 (Roche).

### Adenoviral infection

HUVECs were infected with an AdV-IκBα, an AdV-dnIKK2, or control adenovirus (AdV-GFP), as described previously^18^,^21^,^50^. In order to demonstrate the efficiency of IκBα overexpression, we have measured the expression of total IκBα protein in control cells and cells infected with adenoviral vectors for overexpression of IκBα (AdV-IκBα) using IκBα InstantOne™ ELISA (Affymetrix eBioscience, San Diego, CA, USA).

### Rotational thromboelastometry measurement (ROTEM)

To investigate whether the IL-33-induced TF in ECs could affect the coagulation capacity of human whole blood and plasma, ROTEM (TEM Innovations, Munich, Germany) assay was used, as described previously^25^. HUVECs were seeded onto collagen-coated Cytodex 3 micro-beads (GE Healthcare Biosciences AB, Uppsala, Sweden) and incubated overnight^25^. HUVECs-coated micro-beads were then incubated with or without 100 ng/mL rh IL-33 for 4 h in the presence or absence of 5 μg/mL sST2. LPS (Sigma) at 1 μg/mL was used as positive control. Uncoated micro-beads were used as negative control. Blood from healthy volunteers (n = 3) was collected in 3 separate 3.2% trisodium citrate tubes immediately before ROTEM analysis. The first tube was discarded, and one of the two remaining tubes was centrifuged at 2500 g and room temperature for 20 min to obtain citrated plasma samples. Lyophilized FVII-deficient plasma (Siemens, Erlangen, Germany) was resuspended in 1 ml of distilled water according to the manufacturer’s instructions. For each condition, 300 μL of citrated whole blood, plasma or FVII-deficient plasma was added into the ROTEM cups. Immediately thereafter, coagulation was initiated by adding CaCl_2_ and HUVECs-coated micro-beads or uncoated micro-beads. The CT in seconds was determined.

### Immunofluorescence analysis of IL-33 and TF in human atherosclerotic plaques

Human carotid atherosclerotic plaques were fixed in 4% formaldehyde and embedded in paraffin. Immunofluorescence analysis of atherosclerotic plaques was performed as recently described^18^,^21^,^51^. We used the following antibodies: mouse monoclonal anti-IL-33 antibody (clone Nessy-1, 1:500 dilution; Enzo Life Sciences, Farmingdale, NY, USA), a rabbit polyclonal anti-vWF antibody (1:500 dilution; Dako, Glostrup, Denmark), a goat polyclonal anti-TF antibody (1:15 dilution; R&D Systems), a mouse monoclonal anti-human α-SMA antibody, (1:100 dilution, clone 1A4, Dako) and a rabbit polyclonal anti-FXIIIa antibody (1:500 dilution, Pierce Antibodies, Thermo Fisher Scientific, Rockford, IL, USA) as well as respective isotype IgG controls (Sigma). All antibodies were diluted in PBS containing 0.05% Tween-20, 3% BSA for blocking and 0.1% Triton X-100 for permeabilization. Nuclear counter staining was performed with DAPI (1 μg/mL; Sigma) for 10 minutes at room temperature. Tissue sections were analysed with a confocal laser scanning microscope (LSM-780; Carl Zeiss) using ZEN software.

### Statistical analysis

Values are expressed as mean ± SD. Data were compared by ANOVA and t-test. For tissue mRNA correlation, Pearson’s correlation coefficient was calculated using SPSS 21.0 statistical package for Windows. Values of p ≤ 0.05 were considered significant.

## Additional Information

**How to cite this article**: Stojkovic, S. *et al.* Tissue factor is induced by interleukin-33 in human endothelial cells: a new link between coagulation and inflammation. *Sci. Rep.* **6**, 25171; doi: 10.1038/srep25171 (2016).

## Supplementary Material

Supplementary Information
