# Supplementary material for: Tissue factor is induced by interleukin-33 in human endothelial cells: a new link between coagulation and inflammation
Source: Sci Rep. 2016 May 4;6:25171. doi: 10.1038/srep25171 (PMC4855148; doi:10.1038/srep25171)
Supplement: Supplementary Information [file srep25171-s1.pdf]

Supplementary information

Tissue factor is induced by interleukin-33 in human endothelial cells: a new link between coagulation and inflammation

Stefan Stojkovic[1,2], Christoph Kaun[1], Jose Basilio[3], Sabine Rauscher[4], Lena Hell[5], Konstantin A. Krychtiuk[1], Cornelia Bonstingl[6], Rainer de Martin[3], Marion Gröger[4], Cihan Ay[5], Wolfgang Holnthoner[6,7], Wolfgang Eppel[8], Christoph Neumayer[9], Ihor Huk[9], Kurt Huber[10], Svitlana Demyanets[11,*], Johann Wojta[1,2,4]

- Supplementary Table
- Supplementary Figures

Supplementary Table. Primers and universal probe library (UPL) probes used for qRT-PCR

Target gene	Forward primer	Reverse primer	UPL probe #	Amplicon size [bp]
GAPDH	5’-agccacatcgctcagacac-3’	5’-gcccaatacgaccaaatcc-3’	60	66
TF	5’-cagacagcccggtagagtgt -3’	5’-ccacagctccaatgatgtagaa -3’	2	75
TFPI	5’-gcctgggcaatatgaacaat-3’	5’-ccacctggaaaccattcg-3’	47	81

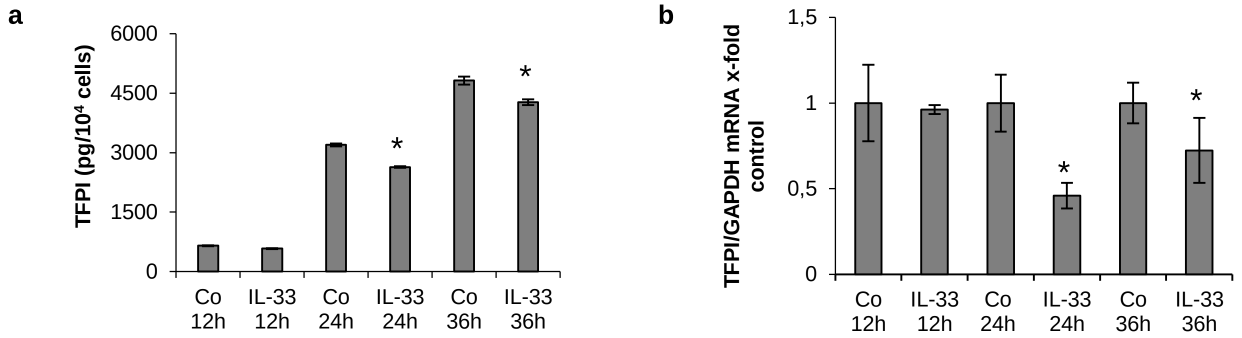

Supplementary Figure S1. IL-33 downregulates tissue factor pathway inhibitor (TFPI) mRNA and protein expression in human endothelial cells.

a, b HUVEC were incubated for 12, 24, or 36 h in the absence (control, Co) or presence of IL-33 (100 ng/mL). After the incubation, supernatant was collected and TFPI protein was determined (a), or mRNA was prepared and RealTime-PCR for TFPI and glyceraldehyde-3-phosphate-dehydrogenase (GAPDH) was performed (b). Values are given in pg/10^4 cells (a) or as TFPI/GAPDH mRNA x-fold change from respective control, which was set as 1 (b), and represent mean values ± SD of three independent determinations. *$p \leq 0.05$ compared to respective control. Each experiment was performed three times. A representative experiment is shown.

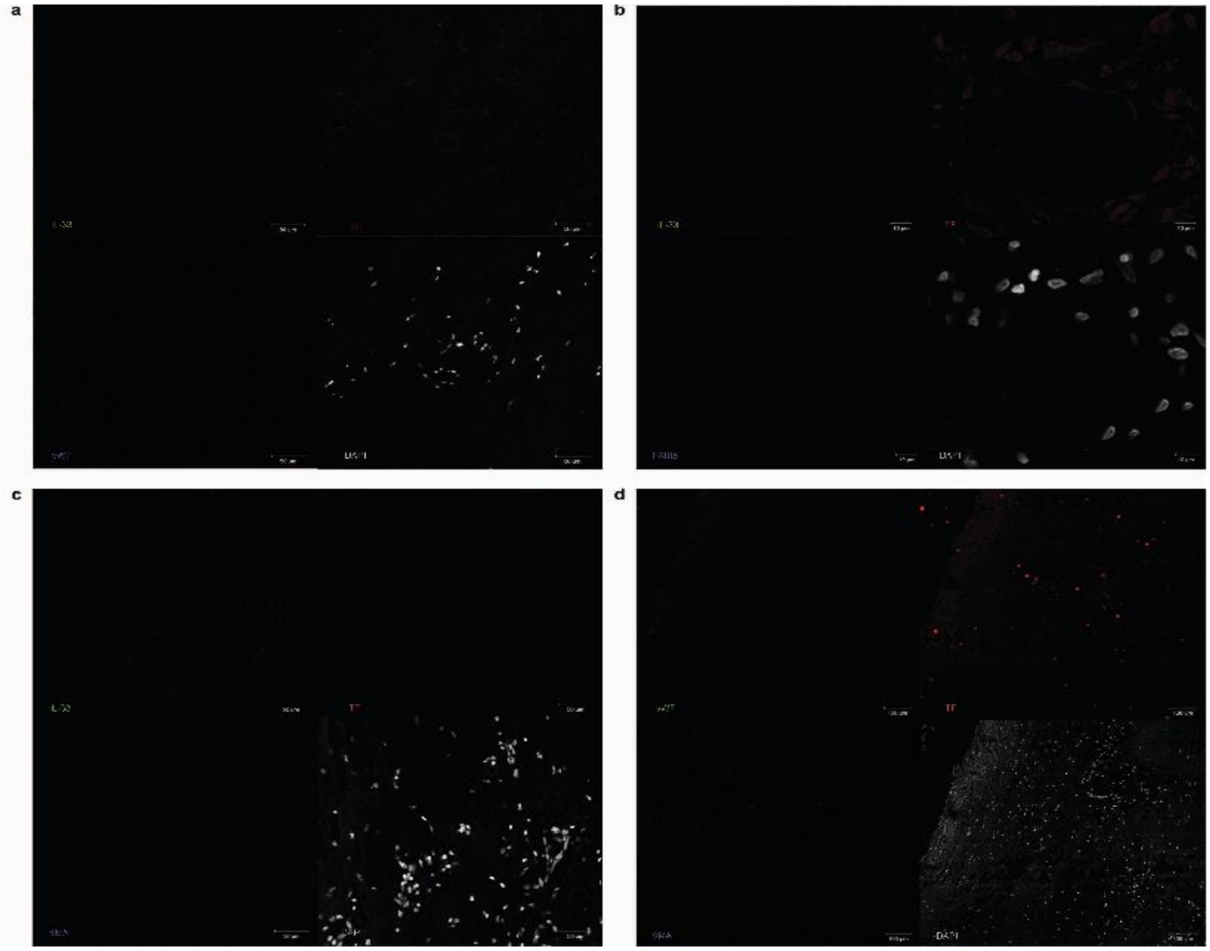

Supplementary Figure S2. Isotype controls of antibodies used in immunofluorescence analysis of human atherosclerotic plaques.

a, b, c, d represent the consecutive sections of the same atherosclerotic plaque as shown in figure 6. **a**, Mouse IgG isotype control (green, IL-33 in Fig. 6a), goat IgG (red, TF in Fig. 6a), rabbit IgG (blue, vWF in Fig. 6a); **b,** mouse IgG isotype control (green, IL-33 in Fig. 6b), goat IgG (red, TF in Fig. 6b), rabbit IgG (blue, FXIIIa in Fig. 6b); **c,** mouse IgG isotype control (green, IL-33 in Fig. 6c), goat IgG (red, TF in Fig. 6c), mouse IgG (blue, SMA in Fig. 6c); **d,** rabbit IgG isotype control (green, vWF in Fig. 6d), goat IgG (red, TF in Fig. 6d), mouse IgG (blue, SMA in Fig. 6d). Original magnification ×63 (a, b, c) or x10 (d). Staining was performed with atherosclerotic samples from 4 different donors. Representative pictures are shown.
